# The lack of BTK does not impair monocytes and polymorphonuclear cells functions in X-linked agammaglobulinemia under treatment with intravenous immunoglobulin replacement

**DOI:** 10.1371/journal.pone.0175961

**Published:** 2017-04-19

**Authors:** Filomena Monica Cavaliere, Alessandro Prezzo, Caterina Bilotta, Metello Iacobini, Isabella Quinti

**Affiliations:** 1 Department of Molecular Medicine, Sapienza University of Rome, Roma, Italy; 2 Department of Pediatrics, Sapienza University of Rome, Roma, Italy; "INSERM", FRANCE

## Abstract

The lack of BTK in X-linked agammaglobulinemia (XLA) patients does not affect monocytes and polymorphonuclear cells (PMN) phenotype and functions. In this study, we show that XLA patients had an increased frequency of the intermediate monocytes subset and that BTK-deficient monocytes and PMN had a normal expression of receptors involved in the activation and cellular responses. We demonstrate that BTK is not required for migration, phagocytosis and the production of reactive oxygen species (ROS) following engagement of FC gamma receptors (FcγR). XLA monocytes and PMN showed an efficient calcium (Ca^2+^)-independent activation of oxidative burst, suggesting that oxidative burst is less dependent by Ca^2+^ mobilization. The phagocytosis was functional and it remained unaltered also after Ca^2+^ chelation, confirming the independence of phagocytosis on Ca^2+^ mobilization. Intravenous immunoglobulin (IVIg) infusion exerted an anti-inflammatory effect by reducing the frequency of pro-inflammatory monocytes. In monocytes, the IVIg reduce the oxidative burst and phagocytosis even if these functions remained efficient.

## Introduction

X-linked agammaglobulinemia (XLA) is a symptomatic primary antibody deficiency (PAD) caused by mutations in the Bruton’s tyrosine kinase (*BTK*) gene located on the long arm of X-chromosome encoding the cytoplasmic BTK [1, 2]. These mutations lead to the nearly complete arrest of B cell differentiation in the bone marrow at the pre-B cell stage resulting in absent or very low peripheral B cells, agammaglobulinemia, absence of B cell-dependent lymphoid tissues and inability to mount specific antibody responses [3–5]. BTK is involved in regulation of proliferation, differentiation and survival of B cells and in functions of innate immune cells, as shown by the altered innate immune responses observed in monocytes, dendritic cells (DC) and neutrophils of XLA patients [6–9]. BTK is a mediator acting downstream the spleen tyrosine kinase (Syk), a cytoplasmic protein-tyrosine kinase member of Src family of non-receptor tyrosine kinases. BTK is normally activated by a variety of receptors, included Fc gamma receptors (FcγR). The antigen engagement of the FcγRs is able to induce the activation of Syk [10–12] that consequently activates the axis BTK-PLCγ2/Ca^2+^ signaling pathway [13, 14].

XLA patients develop during the first year of life recurrent bacterial infections, mostly in the respiratory tract, gastrointestinal tract and skin [15, 16]. Beside the absence of B cells, XLA patients occasionally present neutropenia suggesting a potential neutrophil dysfunction [17], as also demonstrated in the mouse model of XLA [18]. The standard therapy in XLA is the administration of human polyvalent immunoglobulins G (IgG) [19, 20] that beside their antibody replacement activity have been shown to modulate innate immune cells [21, 22]. We have previously shown that in patients affected by a different PAD entity, the Common Variable Immune Disorders (CVID) [23], the IgG administration reduced the overexpression of CD11b and of sialic acid-binding immunoglobulin-like lectin receptor (Siglec 9) on monocytes and the frequency of peripheral pro-inflammatory monocytes. The expansion of pro-inflammatory monocytes has been observed also in other systemic inflammatory diseases [24, 25] together with an overexpression of CD11c, a receptor involved in adherence and phagocytosis [26, 27]. In XLA patients, we evaluated the expression of CD181, CD66b, CD11b, CD11c, CD16 and Siglec 9 receptors on unstimulated and *E*. *coli*-stimulated monocytes and PMN. CD181 (also called CXCR1) is a surface receptor for IL-8, a primary chemo-attractant involved in innate immune cells activation [28]. CD66b is exclusively expressed on human granulocytes and it is recognized as a granulocyte activation marker, involved in neutrophils adhesion, migration and pathogen binding [29, 30]. CD11b is a component of the phagocytic receptor αMβ2 (or CD11b/CD18) involved in phagocytosis, expressed on human PMN, NK cells and mononuclear phagocytes. Siglec 9 is a member of transmembrane sialic acid-binding proteins CD33-related [31, 32] with a postulated inhibitory activity on the immune response through host and bacterial sialoglycans recognition [33, 34]. The simultaneous expression of CD11b and Siglec 9 might play a pivotal role in monocytes activity [23]. CD11c is an integrin molecule, which is a member of αXβ2 receptor that overlap the properties of αMβ2 integrin involved in adherence and phagocytosis of complement coated particles [26, 27]. The clearance of invading microorganisms by innate immune cells constitutes an essential arm of host defense. The entire process can be separated in several stages: adhesion, migration into inflamed tissues, phagocytosis and oxidative burst. Since Ca^2+^ mobilization is involved in ROS production through the axis BTK-PLCγ2, induced by FcγR clustering [35, 36], it is possible that the *BTK* mutation in XLA patients could affect the calcium flux from internal storage, impairing the ROS production. Thus, we verified if the lack of BTK kinase activity affect monocytes and PMN phagocytosis, oxidative burst and Ca^2+^ mobilization. In addition, since BTK is involved in degranulation and in FcγR-mediated cytokine production [36], we analyzed additional PMN functions that might be impaired in XLA including IL-8 production and elastase release. Finally, PMN and monocytes frequencies and functions were analyzed *ex vivo* shortly after intravenous IgG (IVIg) infusions administered at replacement dosages.

## Materials and methods

### Patients and controls

XLA was diagnosed according to the International Union of Immunological Societies Expert Committee for Primary Immunodeficiency criteria [37]. Six adult XLA patients (age range of 20–60 years; mean age: 36.7 ± 15.4 years) and ten age-matched male healthy donors (HD) (age range of 27–58 years; mean age: 39.6 ± 9.8 years) were enrolled for the study. All XLA patients were on replacement treatment, with a cumulative monthly dosage of 400–600 mg/kg of IVIg administered every three weeks (S1 Table). The infusion time ranged from 2 to 3 hours. The infusion speed was established according to the individual tolerability. This study was approved by the Ethics Committee of the Sapienza University of Rome. All participants gave written informed consent prior to inclusion.

### Blood samples preparation

Heparinized whole blood samples were collected from 10 HD and 6 XLA patients immediately before and one hour after IVIg administration. These time points were chosen based on our previous observations [23, 38], taking into consideration that the highest increase of several cytokines plasma concentration occurs within one hour after IVIg infusion [39]. Total peripheral blood monocytes and neutrophils count were determined from blood cell counts and white blood cell differentials. For evaluating circulating cell without the harm of cell loss related to the density gradient centrifugation procedure, peripheral red blood cells was lysed using lysing buffer (Becton Dickinson, BD). Samples were washed twice before staining with various combinations of fluorochrome-labeled antibodies. All antibodies were obtained from BD Biosciences. Flow cytometric analysis was done with a FACSCalibur instrument (BD) using CellQuest (BD) and FlowJo (TreeStar, Ashland, Ore) software. The cytometer stability and sensitivity were checked before each acquisition session by using microbeads designed to control the efficiency, the coefficient of variation of scatter and fluorescence signals and the time delay calibration (Nile Red Fluorescent particles and Calibrite APC Beads, all from BD). Results were expressed as geometric mean Fluorescence Intensity (MFI) of any given marker within the defined population. 30.000 events were counted per sample.

### Phenotypic analysis of monocyte subpopulations

Whole blood samples were first treated to lyse red blood cells and then washed twice and stained at 4°C for 30 min with combinations of fluorochrome-labeled antibodies. Samples were washed, suspended in ice cold PBS and analyzed by a 4-color flow cytometry single platform. Monocytes subpopulations were phenotypically selected by gating on CD14^+^ HLA-DR^+^ monocytes and then classified according to their expression of CD14 and CD16 into classical (CD14^++^CD16^-^), intermediate (CD14^++^CD16^+^) and non classical monocytes (CD14^+^CD16^++^). An isotype control (IgG_1_, BD) with the same fluorochrome of CD16 antibody was run in parallel in order to set the boundary between classical and intermediate monocytes [23]. The surface expression of CD181, CD11b, CD11c and Siglec 9 receptors was evaluated on monocytes samples from erythrocytes-lysed whole blood, performing a staining at 4°C for 30 min with combinations of fluorochrome-labeled antibodies. In parallel, we used an isotype control (IgG_1_, BD) for each receptor analyzed. Results were expressed as percentage of cells that stained positive for a given marker.

### Analysis of receptors expression on PMN

The expression of CD181, CD66b, CD11b, CD11c, CD16 and Siglec 9 was evaluated on PMN samples from erythrocytes-lysed whole blood, performing a staining at 4°C for 30 min with combinations of fluorochrome-labeled antibodies. Samples were washed, suspended in ice cold PBS and analyzed by flow cytometry. PMN were identified by forward scatter (FSC) and side scatter (SSC) characteristics (Panels A and B in S1 Fig).

### Monocytes and PMN stimulation by *Escherichia coli*

One hundred μL of whole blood were added to 20 μl of pre-cooled opsonized not labeled whole *Escherichia coli* (*E*. *coli*) at a concentration of 1-2x10^9^/ml (Glycotope, Biotechnology). Samples were incubated in water bath for 20 min at 37°C and erythrocytes were lysed for 15 min at room temperature. Cells stimulated were stained at 4°C for 30 min with fluorochrome-labeled antibodies in various combinations to evaluate the receptors expression.

### Monocytes and PMN oxidative burst activity

The leukocyte oxidative burst was analyzed by using the PHAGOBURST assay (Glycotope, Biotechnology). Whole blood samples (100 μl) were incubated in a water bath for 20 min at 37°C with opsonized *E*. *coli* (1-2x10^9^/ml), PMA (1.62 mM) and wash solution as negative control. The percentage and MFI of cells that produced ROS was analyzed. The intracellular production of superoxide anions and hydrogen peroxide in monocytes and PMN in response to phagocytosis of bacteria was tested by using the fluorescence probes dihydrorhodamine 123 (DHR 123). We repeated the analysis of the oxidative burst using non-opsonized *E*. *coli* (1.5x10^9^/ml) as control in order to exclude the FcγR-independent phagocytosis. In parallel, we performed the study of oxidative burst by using BAPTA-AM (Unimed Scientifica) as calcium chelator, in order to verify the Ca^2+^-dependence production of ROS triggered by FcγR stimulation. Whole blood samples were pre-treated with 100 μM of BAPTA-AM for 30 min and then they were incubated in a water bath for 20 min at 37°C with opsonized *E*. *coli* (1-2x10^9^/ml). The oxidation lead to fluorescence detected by flow cytometry, by using the blue-green excitation light (488 nm argon-ion laser).

### Monocytes and PMN phagocytosis

The leukocyte phagocytosis was determined by using a PHAGOTEST assay (Glycotope, Biotechnology). Whole blood samples (100 μl) were incubated in a water bath for 10 min at 37°C with opsonized FITC-labeled *E*. *coli* (2x10^9^/ml). As negative controls, 100 μl of each sample were incubated in an ice bath for 10 min with opsonized FITC-labeled *E*. *coli* (2x10^9^/ml). As control, the same experiment was done by using non opsonized FITC-labeled *E*. *coli* (2x10^9^/ml). The percentage and MFI of granulocytes and monocytes having performed phagocytosis was analyzed. In parallel, we tested the effect of calcium chelator on phagocytosis in order to confirm the Ca^2+^-independence of the process. Whole blood samples were pre-treated with 100 μM of BAPTA-AM for 30 min and then incubated in a water bath for 10 min at 37°C with FITC-labeled *E*. *coli* (2x10^9^/ml). Cells were analyzed by flow cytometry by using the blue-green excitation light (488 nm argon-ion laser).

### PMN migration

PMN chemotaxis was determined by the MIGRATEST CHEMOTAXIS assay (Glycotope, Biotechnology). Heparinized whole blood samples (1000 μl) were added to leukocyte separation medium for isolation of leukocyte-rich plasma (LRP), for 40 min at room temperature. After isolation, 100 μl of LRP were added into cell culture insert (3.0 μm pore size) and incubated in a water bath for 30 min at 37°C with chemotactic peptide N-formyl-Met-Leu-Phe (fLMP). After incubation, the cell suspensions were added in two separate tubes and placed on ice. 20 μl of cell suspension from the cell culture insert of the negative control were mixed with 180 μl of incubation buffer and then transferred on a third tube. 20 μl of antibody and counting reagent were added to LRP and incubated for 10 min in an ice bath in the dark. Finally, 20 μl of vital DNA staining solution were added per tube and incubated 5 min on ice. The number of migrated PMN was analyzed by flow cytometry by using the blue-green excitation light (488 nm argon-ion laser).

### PMN elastase assay

The PMN elastase was quantified by Human PMN Elastase Platinum ELISA (Affymetrix eBioscience). 100 μl of each plasma sample were added to a microwell plate coated with polyclonal antibody to human PMN elastase and incubated at room temperature for 1 hour. After washing, samples were incubated with TMB Substrate Solution at room temperature for 20 min in the dark. 50 μl of stop solution were added to stop the reaction. The absorbance was determined at 450 nm on a microplate reader (Multiskan^™^ FC Microplate Photometer, Thermo Scientific). Concentrations were expressed as ng/ml.

### IL-8 plasma dosage

Interleukin-8 (IL-8) was measured by using a commercially available kit ELISA (CliniSciences). Plasma was collected from blood samples unstimulated and stimulated with *E*. *coli* by centrifugation for 15 min at 1000 x g, diluted and tested in duplicates.

100 μl of each plasma sample were added to a microwell plate coated with monoclonal antibody to human IL-8 and incubated at 37°C for 90 min. After washing, samples were incubated with Biotin-Conjugate Solution at 37°C for 1 h. Then, each well was washed several times and incubated with 100 μl of Streptavidin-HRP at 37°C for 30 min. After washing, 100 μl of Substrate Solution were added to each well and incubated for 10–20 min a 37°C. 100 μl of stop solution were added to stop the reaction. The absorbance was determined at 450 nm on a microplate reader (Multiskan^™^ FC Microplate Photometer, Thermo Scientific). Concentrations were expressed as pg/ml.

### Statistical analysis

Data were analyzed using the Mann-Whitney U test for unpaired two groups or by paired Wilcoxon test. Correlations were calculated by means of linear regression analysis.

Data were analyzed with StatView 5.0.1 software (SAS Institute, Cary, NC). A p value equal or less than 0.05 was considered as statistically significant.

## Results

### Monocyte subsets

**Fig 1:**
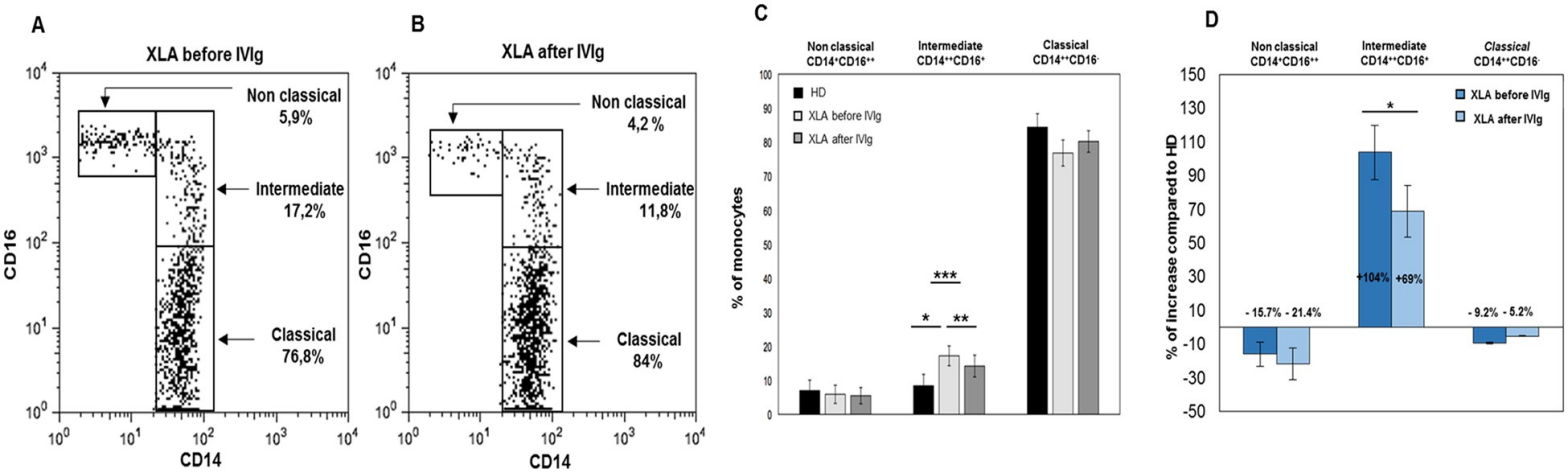
Frequencies of non classical, intermediate and classical monocytes increased from XLA patients before and after IVIg infusion respect to HD. (A and B) Monocytes subpopulations were phenotypically classified according to their expression of CD14 and CD16 into classical (CD14^++^CD16^-^), intermediate (CD14^++^CD16^+^) and non classical monocytes (CD14^+^CD16^++^) in a representative XLA patient before and after IVIg infusion. Percentages denote mean values. (C) Histograms show that non classical and classical monocyte frequencies from XLA patients are similar to that observed on HD and that IVIg infusion did not change their frequency. Intermediate monocytes percentage is increased in XLA patients (▪p = 0.01), IVIg infusion induce their reduction (▪▪p = 0.04) even if remained at higher level respect to HD (▪▪▪p = 0.01). (D) Histograms denote the percentage of increase of the three monocytes subsets of XLA patients respect to HD. Histograms denote mean values and bars standard deviation. Statistical significance as determined by the nonparametric Mann Whitney and Wilcoxon Signed Rank test is indicated as *p* value.

Monocytes subsets were identified according to their expression CD14 and the CD16 (Fig 1A and 1B). Absolute counts of peripheral blood total monocytes, classical and non classical monocytes did not show any difference between XLA and HD. Classical and non classical monocyte were similarly represented in XLA and HD while the frequency of intermediate monocytes was increased in XLA (Fig 1C). In Fig 1D we show the increase of monocytes subsets from XLA patients compared to HD expressed as percentage.

After IVIg, the frequency of intermediate monocytes rapidly decreased remaining higher than HD (Fig 1C and 1D) while the frequency of non classical and classical monocyte subsets did not change (Fig 1C).

### Receptors expression

#### Monocytes

**Fig 2:**
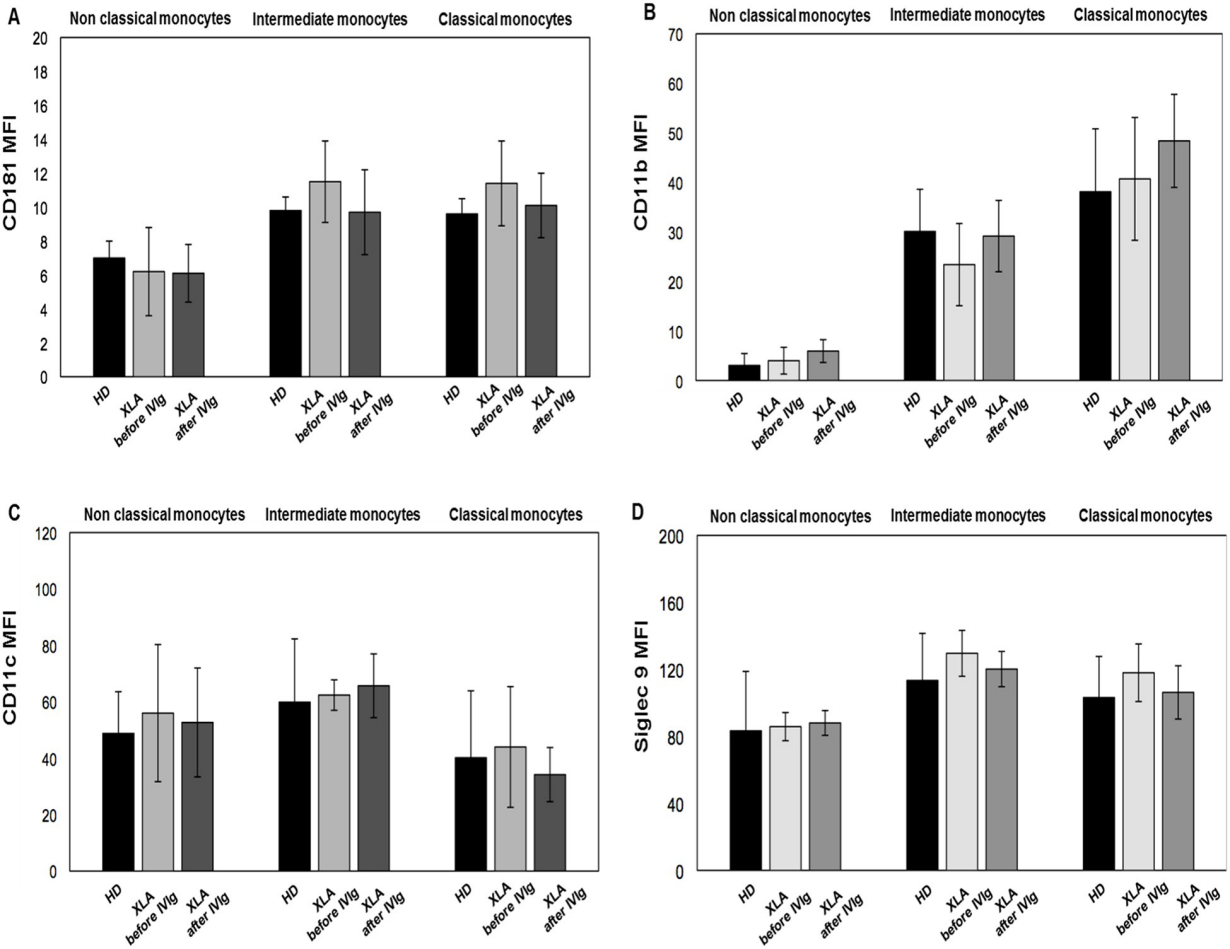
CD181, CD11b, CD11c and Siglec 9 expression on monocytes subsets from HD and XLA patients before and after IVIg infusion. Whole blood samples were analyzed for the expression of CD181, CD11b, CD11c and Siglec 9 before and after IVIg infusion. The expression of all surface receptors was evaluated by performing a staining at 4°C for 30 min with specific fluorochrome-labeled antibody. Samples were washed, suspended in ice-cold PBS and analyzed by flow cytometry. XLA patients show a similar CD181, CD11b, CD11c and Siglec 9 expression on monocytes subsets compared to HD. After IVIg infusion the expression of all receptors on monocytes subsets remained unaltered. Results are expressed as Mean Fluorescence Intensity. Histograms denote mean values and bars standard deviation.

We evaluated the surface expression of CD181, CD11b, CD11c and Siglec 9 receptors involved in the regulation of monocytes function. As shown in Table 1, we found that all receptors were expressed at a similar level in the three monocytes subsets in XLA patients and HD. Moreover, IVIg administration did not induce any variation of their expression (Fig 2A and 2D). The stimulation with opsonized *E*. *coli* induced an increase of all receptors similarly in XLA patients and HD (Table 1). This overexpression induced by *E*. *coli* was preserved after IVIg, showing that IVIg replacement did not affect the monocytes ability to respond to opsonized bacteria (Table 1).

**Table 1:** Receptors expression on monocytes subsets from HD and XLA patients.

		HD			XLA before IVIg			XLA after IVIg		
Receptor		Non classical	Intermediate	Classical	Non classical	Intermediate	Classical	Non classical	Intermediate	Classical
**CD181 (MFI)**	**UN**	7.1 ± 1	9.8 ± 2.6	9.6 ± 1.7	6.2 ± 0.8	11.5 ± 2.4	11.4 ± 2.5	6.1 ± 0.9	9.7 ± 2.5	10.1 ± 1.9
	**ST**	22.3 ± 1.5	15.2 ± 3.6	15.6 ± 1.4	18.6 ± 1.4	17.2 ± 3.5	17.8 ± 2.7	18.4 ± 1.9	13.9 ± 4.7	15.1 ± 2.2
**CD11b (MFI)**	**UN**	3 ± 2.4	30.1 ± 8.5	38.1 ± 12.7	4 ± 2.7	23.4 ± 8.3	40.7 ± 12.4	5.9 ± 2.3	29.2 ± 7.2	48.4 ± 9.4
	**ST**	8.6 ± 4	72.8 ± 9.8	96.7 ± 12.1	9 ± 6.6	62 ± 25.2	75.5 ± 18.8	14.1 ± 8.6	70.3 ± 27	75.4 ± 26.1
**CD11c (MFI)**	**UN**	40.2 ± 19.3	59.9 ± 24.3	48.8 ± 14.8	56 ± 22.4	62.4 ± 5.4	50.2 ± 10	52.7 ± 23.7	65.7 ± 21.4	38.4 ± 7.7
	**ST**	128.5 ± 10.6	117.3 ± 21.8	101.4 ± 9.5	140.5 ± 0.7	120.5 ± 2.1	111.5 ± 0.7	141 ± 9.9	112 ± 15.6	107 ± 14.1
**Siglec 9 (MFI)**	**UN**	83.3 ± 35.5	113.3 ± 28.1	103.2 ± 24.5	85.8 ± 8.4	129.6 ± 13.7	118 ± 17.2	88 ± 7.4	120.2 ± 10.5	106.3 ± 15.9
	**ST**	102.2 ± 42.6	151.6 ± 38.4	146.5 ± 38.4	138.9 ± 56.5	151.5 ± 22.6	177.8 ± 55.2	96.8 ± 24.2	143.6 ± 27	143.5 ± 34.9

Values are expressed as Mean Fluorescence Intensity (MFI). UN: Unstimulated; ST: Stimulated with *E*. *coli*.

#### PMN

**Fig 3:**
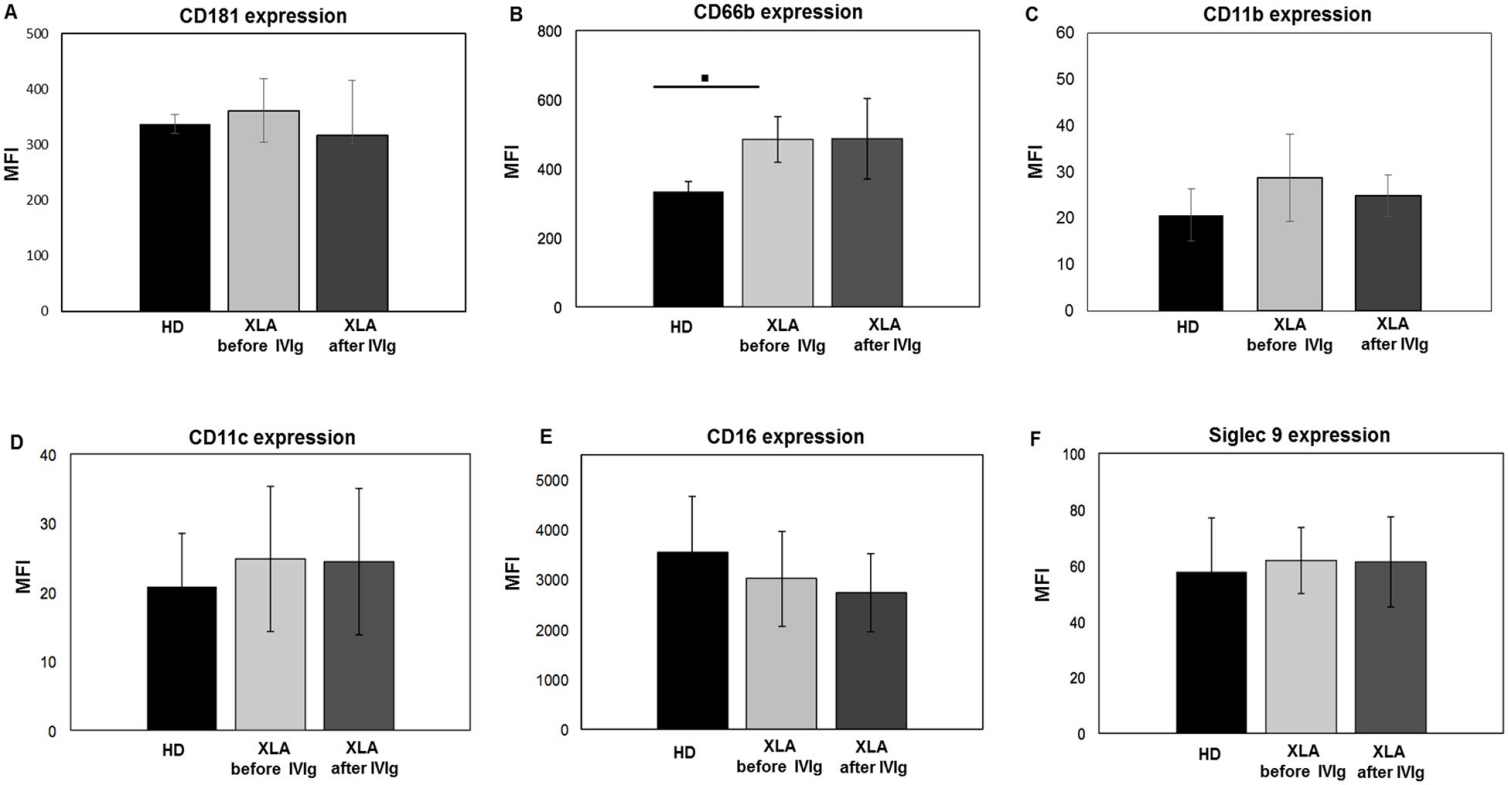
CD181, CD11b, CD11c and Siglec 9 expression on PMN from HD and XLA patients before and after IVIg infusion. Whole blood samples were analyzed for the expression of CD181, CD66b, CD11b, CD11c, CD16 and Siglec 9 before and after IVIg infusion. The expression of all surface receptors was evaluated by performing a staining at 4°C for 30 min with specific fluorochrome-labeled antibody. Samples were washed, suspended in ice-cold PBS and analyzed by flow cytometry. XLA patients and HD show a similar CD181, CD11b, CD11c, CD16 and Siglec 9 expression, while CD66b expression was higher on PMN from XLA patients compared to HD (▪p = 0.001*)*. After IVIg infusion, the expression of all receptors remained unaltered. Results are expressed as Mean Fluorescence Intensity. Histograms denote mean values and bars standard deviation. Statistical significance, determined by the nonparametric Mann Whitney test, is indicated as *p* value.

**Fig 4:**
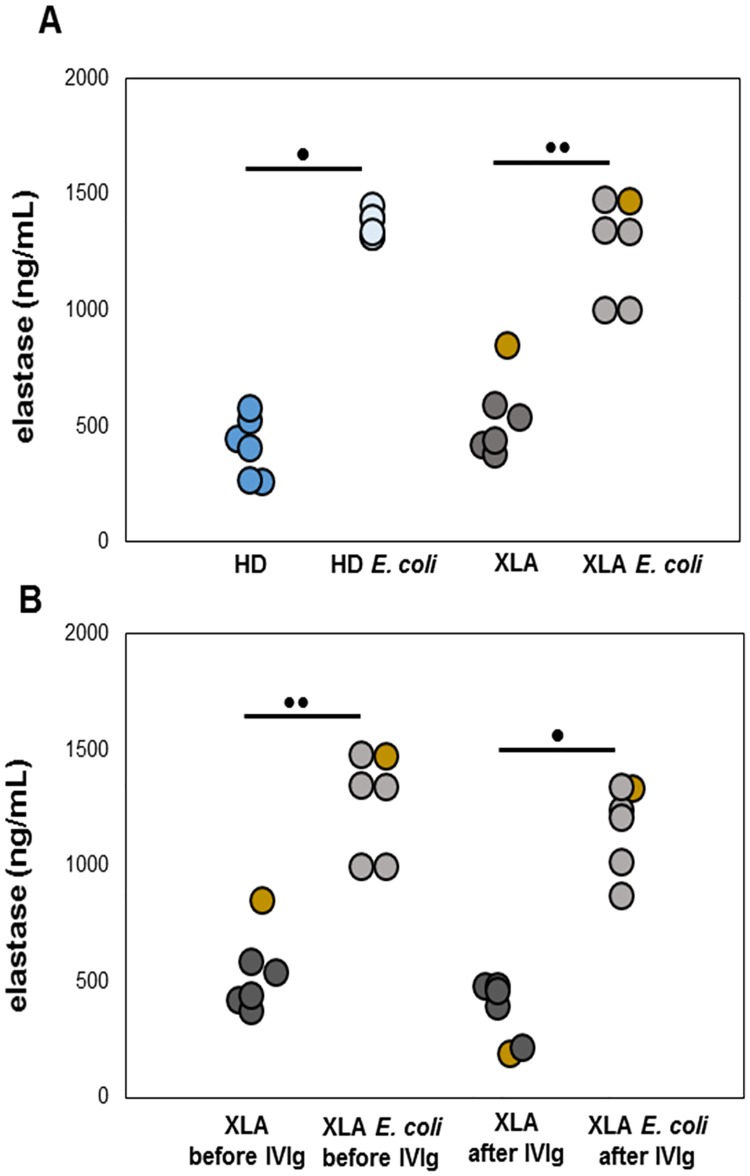
PMN elastase release. The PMN elastase was quantified by ELISA. Plasma samples were added to a microwell plate coated with polyclonal antibody to human PMN elastase and incubated for 1 hour. (A) Data show that, at baseline, the elastase release was similar in XLA and HD and that after *E*. *coli* stimulation, it rapidly increased. (B) After the IVIg infusion, PMN maintained their degranulation ability. Only one patient showed a high PMN elastase release, at baseline (gold). Concentrations were expressed as ng/ml. •p = 0.028; ••p = 0.027.

The activation status of PMN strongly influence the respiratory burst, phagocytosis and adhesion processes. As shown in Fig 3A and 3F and in Table 2, PMN from XLA and HD showed an overlapping expression of all receptors with the exception of CD66b that was overexpressed in XLA patients (Fig 3B). However, this increase was not correlated with a physiological dysfunction, as demonstrated by the normal PMN ability to release elastase, a proteinase needed to degrade foreign organisms and host tissue during inflammation. In fact, at baseline, the elastase release was similar in XLA and HD (Fig 4A and 4B). After *E*. *coli* stimulation, the elastase release rapidly increased in XLA and in HD. After IVIg infusion, PMN maintained their degranulation ability (Fig 4B).

**Table 2:** Receptors expression on PMN from HD and XLA patients.

	HD		XLA before IVIg		XLA after IVIg	
Receptor	Unstimulated MFI	Stimulated MFI	Unstimulated MFI	Stimulated MFI	Unstimulated MFI	Stimulated MFI
**CD181**	337.3 ± 16.8	205.8 ± 102.9*	361.5 ± 57.3	198 ± 76.5**	317.5 ± 98.3	169 ± 69.3**
**CD66b**	333.5 ± 29	1310 ± 162.6*	484.7 ± 66	1365.7 ± 287.7**	486.7 ± 116.4	1240 ± 654**
**CD11b**	20.7 ± 5.7	50.6 ± 0.8*	28.7 ± 9.4	49.3 ± 22.7**	24.8 ± 4.5	51.3 ± 29.5**
**CD11c**	20 ± 7.7	48.7 ± 11*	24.8 ± 10.5	50.4 ± 14.4**	24.4 ± 10.6	47.9 ± 13.6**
**CD16**	3555.6 ± 1144.5	4336.6 ± 1539.5*	3012 ± 952.8	3758.8 ± 1035.2**	2753.8 ± 794.4	3594.6 ± 1033.6**
**Siglec 9**	56.1 ± 15.5	99 ± 38.7*	61.7 ± 11.9	97.2 ± 27.4**	61.2 ± 16.1	81.7 ± 28.1**

Values are expressed as Mean Fluorescence Intensity (MFI).

* p 0.005,

** p 0.03

**Fig 5:**
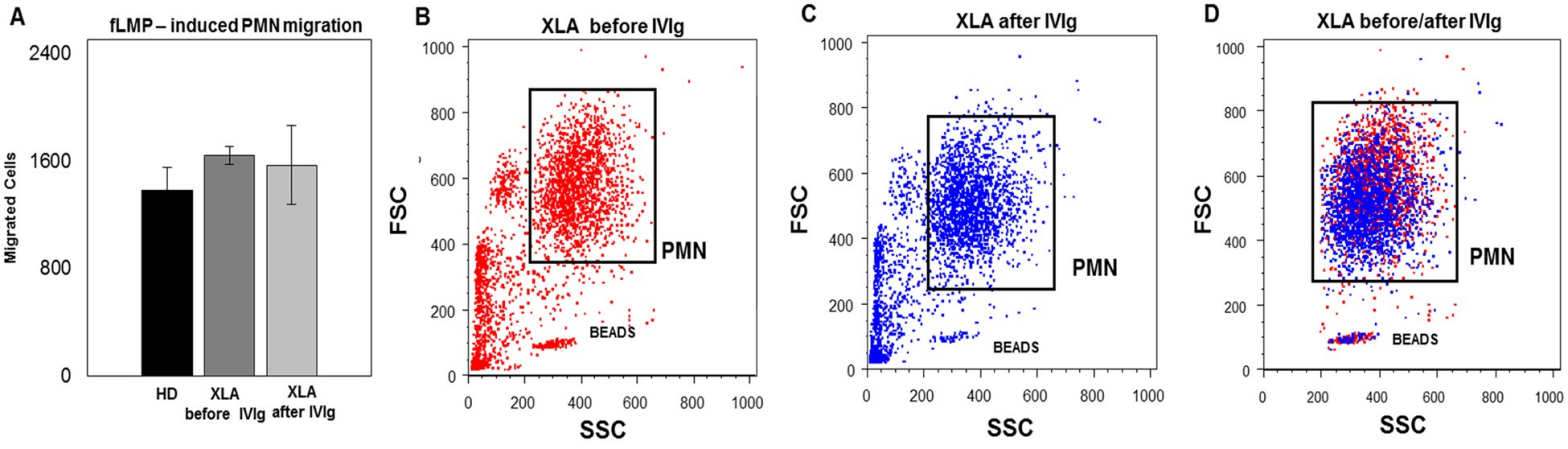
PMN migration. The quantitative determination of PMN chemotaxis was observed by incubating heparinized whole blood samples on leukocyte separation medium for isolation of leukocyte-rich plasma (LRP). After isolation, LRP were incubated in a water bath for 30 min at 37°C with fLMP. Values are expressed as number of PMN which have migrated by flow cytometry. (A) Data show that PMN migration induced by fMLP was comparable in XLA patients and HD. (A) After IVIg, the PMN migration remained unaltered. Histograms denote mean values and bars standard deviation. (B, C and D) An example of the FACS analysis of PMN migration of a representative XLA patient before and after IVIg infusion. The migrated cells were gated and analyzed within the quadrants of the dot plots.

Moreover, also the PMN migration induced by fMLP was comparable in XLA patients and HD. After IVIg, the PMN migration remained unaltered (Fig 5A and 5D). Thus, the explanation for the increased CD66b remained obscure.

*E*. *coli* stimulation induced an overexpression of all receptors in XLA patients and HD, with the exception of CD181, that was reduced, in line with a previous study [28].

After IVIg infusion, the expression of all receptors in unstimulated conditions and their overexpression induced by *E*. *coli* remained unaltered, demonstrating that IVIg did not influence the phenotype of PMN, as previously shown in CVID patients [38] and did not affect the PMN ability to respond to opsonized bacteria (Table 2).

### IL-8 production

**Fig 6:**
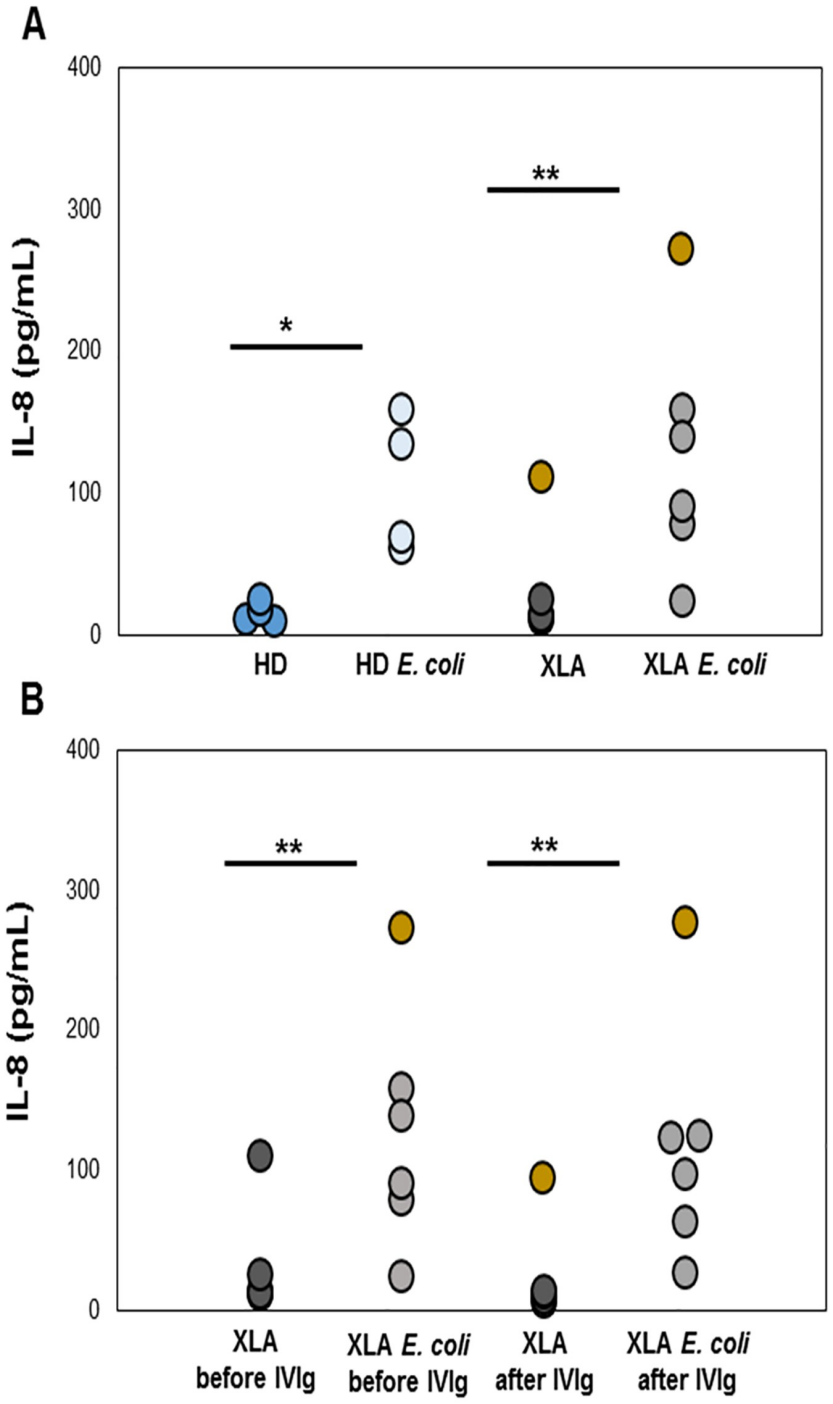
Plasma levels of IL-8 in XLA patients and HD. IL-8 production was measured by using a kit ELISA. IL-8 plasma levels were similar in XLA and HD. (A) After *E*. *Coli* stimulation, IL-8 increased in XLA and HD. (B) After IVIg infusion, IL-8 levels remained unchanged and the level after *E*. *coli* stimulation was well preserved. Data show that only one XLA patient (gold) with a severe inflammatory condition had a very high IL-8 plasma levels. The same patient exhibited high level of IL-8 after *E*. *Coli* stimulation. Concentrations were expressed as pg/ml. *p = 0.042; **p = 0.043.

IL-8 plasma levels were similar in XLA (16.3 ± 5.7 pg/ml) and HD (17 ± 7.2 pg/ml) (Fig 6A). After IVIg infusion, IL-8 levels remained unchanged (11.6 ± 3 pg/ml) (Fig 6B). After *E*. *coli* stimulation, IL-8 increased in XLA (99.5 ± 53.1 pg/ml) and HD (107 ± 48.4 pg/ml). After IVIg infusion, the *E*. *coli*-induced IL-8 production remained high (88.2 ± 42.4). Only one XLA patient with a severe inflammatory condition had a very high IL-8 plasma levels (112 pg/ml before IVIg and 94.6 pg/ml after IVIg) (Fig 6A and 6B). The same patient exhibit high level of IL-8 after *E*. *coli* stimulation (before IVIg: 273.2 pg/ml; after IVIg: 277.8 pg/ml). This high IL-8 production was associated in the same patient with a high PMN elastase release (850 ng/ml).

### Phagocytosis

Phagocytosis contributes to the first line of defense against infection trough the engulfment and destruction of invading microorganisms. It also plays a key role in tissue homeostasis and remodeling.

#### Monocytes

**Fig 7:**
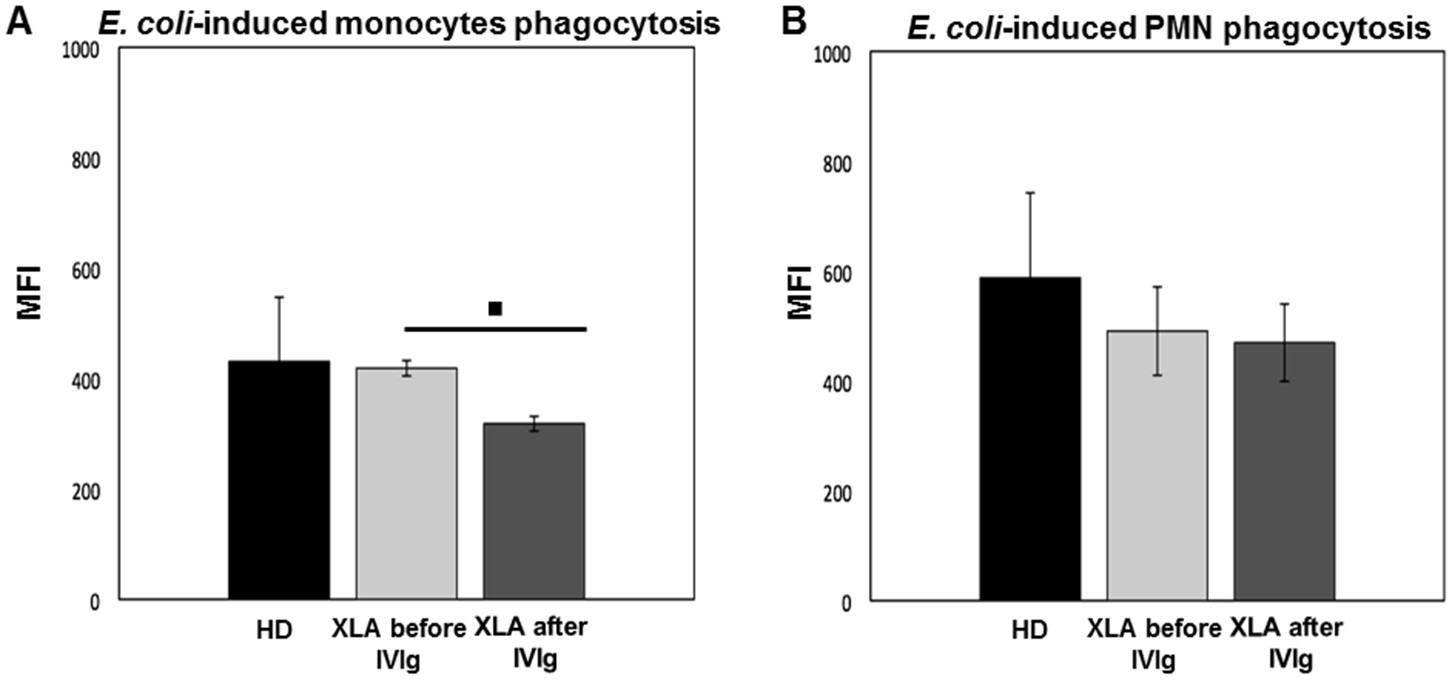
Monocytes and PMN phagocytosis in XLA patients and HD. The quantitative determination of leukocyte phagocytosis was tested by incubating whole blood samples in a water bath for 10 min at 37°C with FITC-labeled *E*. *coli* (2x10^9^/ml). The percentage of cells having performed phagocytosis was analyzed as number of ingested bacteria and results were expressed as Mean Fluorescence Intensity. XLA patients show a similar monocytes phagocytosis compared to HD. (A) After IVIg administration monocytes phagocytosis decreased (▪p = 0.04). XLA patients show a similar PMN phagocytosis compared to HD. (B) After IVIg administration PMN phagocytosis remains unchanged. Histograms denote mean values and bars standard deviation. Statistical significance, determined by the nonparametric Wilcoxon Signed Rank test, is indicated as *p* value.

Monocytes phagocytosis in XLA was normal at baseline (Fig 7A), but it was decreased about 25% after IVIg (Fig 7A). The phagocytosis induced by a non-opsonized *E*. *coli* was similar to the negative control confirming that *E*. *coli* was internalized through a FcγR pathway.

#### PMN

PMN from XLA displayed a normal phagocytosis (Fig 7B). After IVIg, it remained unaltered (Fig 7B), demonstrating that IVIg administered at replacement dosage did not affect the ability of PMN to perform an efficient phagocytosis. Also in PMN, we found that phagocytosis using non-opsonized *E*. *coli* was negligible.

### Oxidative burst

The oxidative burst reflects the killing ability of innate immune cells and it involves many cellular pathways. The lack of BTK activity might influence the respiratory burst, a process requiring Ca^2+^ mobilization from internal storage. We verified this hypothesis on monocytes and neutrophils subsets.

#### Monocytes

**Fig 8:**
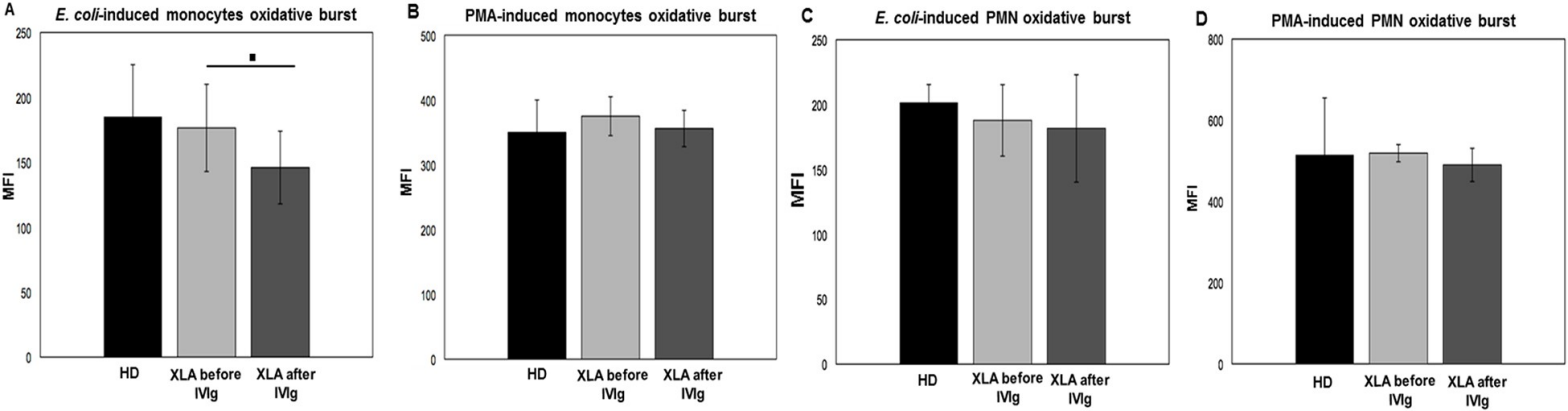
Monocytes and oxidative burst in XLA patients and HD. The quantitative evaluation of leukocyte oxidative burst was observed by incubating whole blood samples in a water bath at 37°C with opsonized *E*. *coli* (1-2x10^9^/ml) or PMA (1.62mM). The conversion of DHR 123 to R 123 were used to evaluated the intracellular ROS production. (A and B) XLA patients show a similar monocytes *E*. *coli*-induced and PMA-induced oxidative burst compared to HD. (C and D) XLA patients show a comparable PMN *E*. *coli*-induced and PMA-induced oxidative burst to HD. After IVIg, monocyte *E*. *coli*-induced oxidative burst slightly decreased (▪p = 0.004), but it remain unaltered when PMA was provided. After IVIg, PMN ROS production remain unaltered. Results are expressed as Mean Fluorescence Intensity. Histograms denote mean values and bars standard deviation. Statistical significance, determined by the nonparametric Wilcoxon Signed Rank test, is indicated as *p* value.

The monocytes ROS production after stimulation with opsonized *E*. *coli* or PMA was comparable in XLA and HD (Fig 8A and 8B). After IVIg infusion, the oxidative burst induced by *E*. *coli* slightly decreased about 13%, while it remained unaltered when PMA was used as stimulus (Fig 8A and 8B). As control, we analyzed the oxidative burst using non-opsonized *E*. *coli* in order to evaluate the oxidative burst resulting from the FcγR-independent phagocytosis. As expected, we found that the oxidative burst was comparable to negative control, confirming that opsonized *E*. *coli* induced the oxidative burst through a FcγRs pathway. The positive correlation between oxidative burst and phagocytosis (Panels A and B in S2 Fig) confirmed the dependence of oxidative burst process from phagocytosis [40–43].

#### PMN

The PMN ROS production after *E*. *coli* and PMA stimulation was comparable in XLA patients and HD (Fig 8C and 8D). After IVIg, the *E*. *coli*-induced and PMA-induced ROS production remained unchanged (Fig 8C and 8D). The PMN oxidative burst induced by non-opsonized E. *coli* was negligible.

All our observations were done in XLA patients under IVIg treatment since many years and thus an interference of this chronic treatment on our data could not be ruled out. In order to verify our findings, we analyzed a newly diagnosed untreated XLA patient (XLA naïve). We found that XLA naïve patient showed receptors expression, migration, phagocytosis and oxidative burst functions similar as XLA patients under chronic IVIg replacement.

### Calcium chelation

The observation that BTK is not essential for the ROS production and that ROS production requires Ca^2+^ mobilization [44, 45] prompted us to verify if lack of expression of functional BTK in XLA patients might be bypassed by alternative mechanisms able to activate Ca^2+^ mobilization or by Ca^2+^-independent mechanisms.

#### Phagocytosis

**Fig 9:**
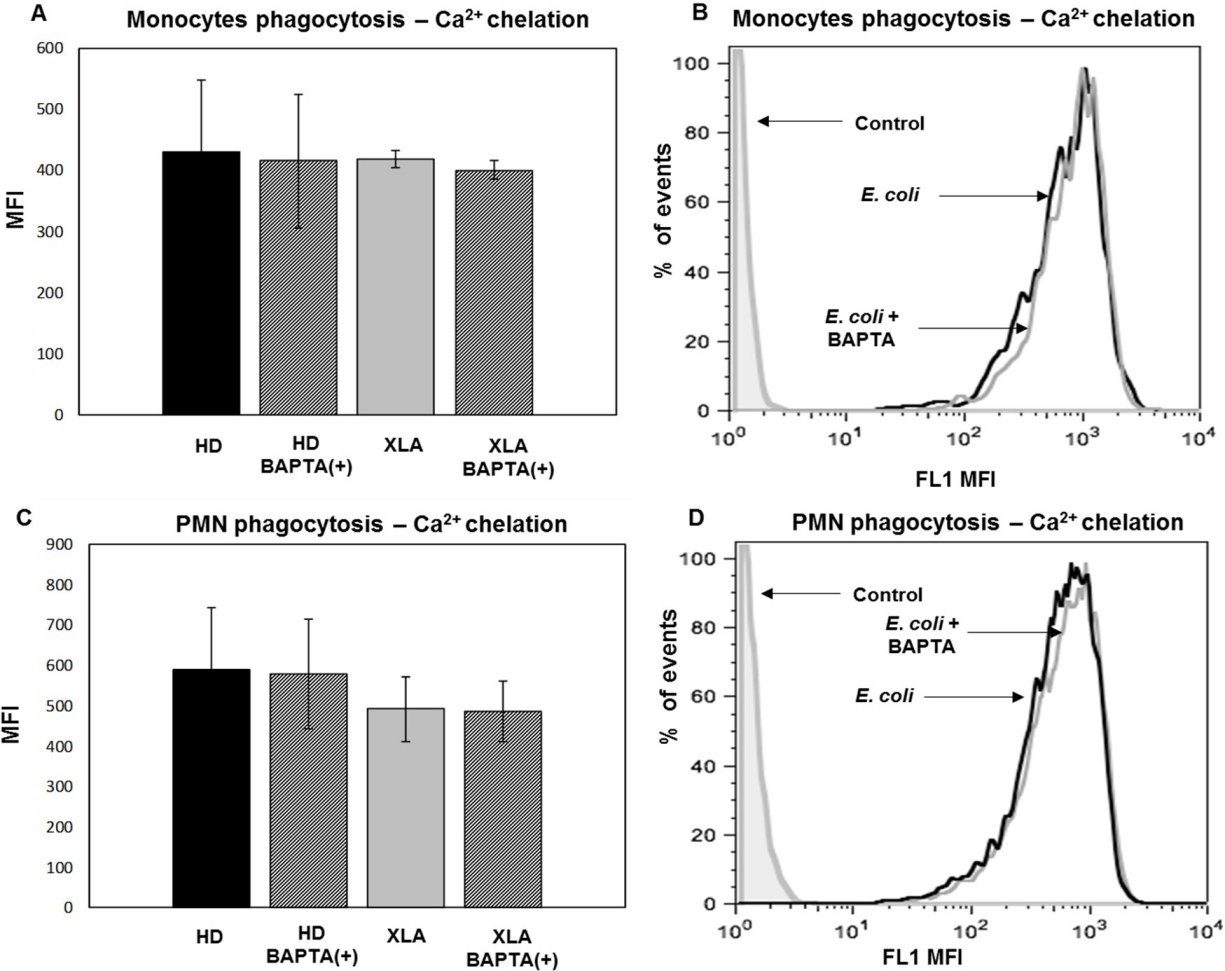
Monocytes and PMN phagocytosis—Ca^2+^ chelation. Whole blood samples were pre-treated with 100 μM of the calcium chelator BAPTA-AM for 30 min and incubated in a water bath for 20 min at 37°C with opsonized *E*. *coli* (1-2x10^9^/ml). The conversion of DHR 123 to R 123 was used to evaluate the intracellular ROS production. FSC and SSC characteristics were used to identify the monocytes population. A regular phagocytosis was observed on monocytes and PMN from HD and XLA even after calcium chelation demonstrating that the process is Ca^2+^independent. Results are expressed as Mean Fluorescence Intensity. Histograms denote mean values and bars standard deviation. In (A and B) are shown monocytes and in (C and D) PMN phagocytosis of representative XLA patient: black peak BAPTA-AM(-) and gray peak BAPTA-AM(+).

To verify the independence of monocytes phagocytosis by Ca^2+^ mobilization [36], in XLA and HD we used the calcium chelator BAPTA-AM. Data confirmed that after calcium chelation the phagocytosis did not changed (Fig 9A and 9B). The independence of phagocytic process by Ca^2+^ mobilization was observed also in PMN (Fig 9C and 9D).

#### Oxidative burst

**Fig 10:**
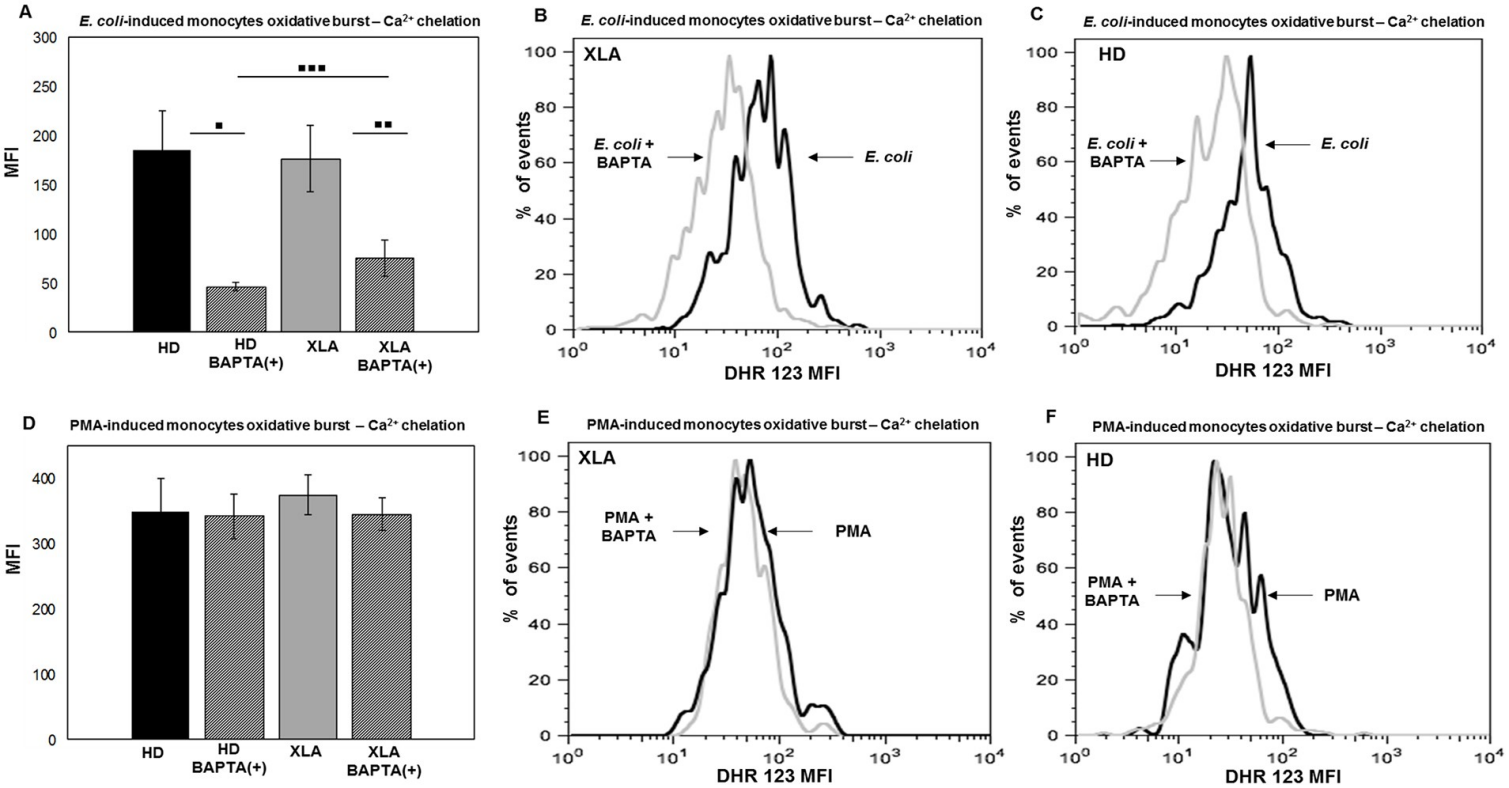
Monocytes oxidative burst—Ca^2+^ chelation. Whole blood samples were pre-treated with 100 μM of the calcium chelator BAPTA-AM for 30 min and incubated in a water bath for 20 min at 37°C with opsonized *E*. *coli* (1-2x10^9^/ml) and PMA (1.62 mM). The conversion of DHR 123 to R 123 was used to evaluate the intracellular ROS production. FSC and SSC characteristics were used to identify the monocytes population. (A) BAPTA-AM treatment cause a stronger reduction of *E*. *coli*-induced oxidative burst in HD than XLA *(*▪▪▪p = 0.003). (A) The average reduction in HD was about 75% (▪p = 0.01) and it was about 50% in XLA patients (▪▪p = 0.02). (D) BAPTA-AM did not affect the oxidative burst induced by PMA both HD and XLA patients. Results are expressed as Mean Fluorescence Intensity. Histograms denote mean values and bars standard deviation. Statistical significance, indicated as p value, was determined by the nonparametric Mann-Whitney test and Wilcoxon Signed Rank test. (B and C) It shown monocytes *E*. *coli*-induced oxidative burst in a representative HD and XLA patient, black peak BAPTA-AM(-) and gray peak BAPTA-AM(+). (E and F) It shown monocytes PMA-induced oxidative burst in a representative HD and XLA patient, black peak BAPTA-AM(-) and gray peak BAPTA-AM(+).

**Fig 11:**
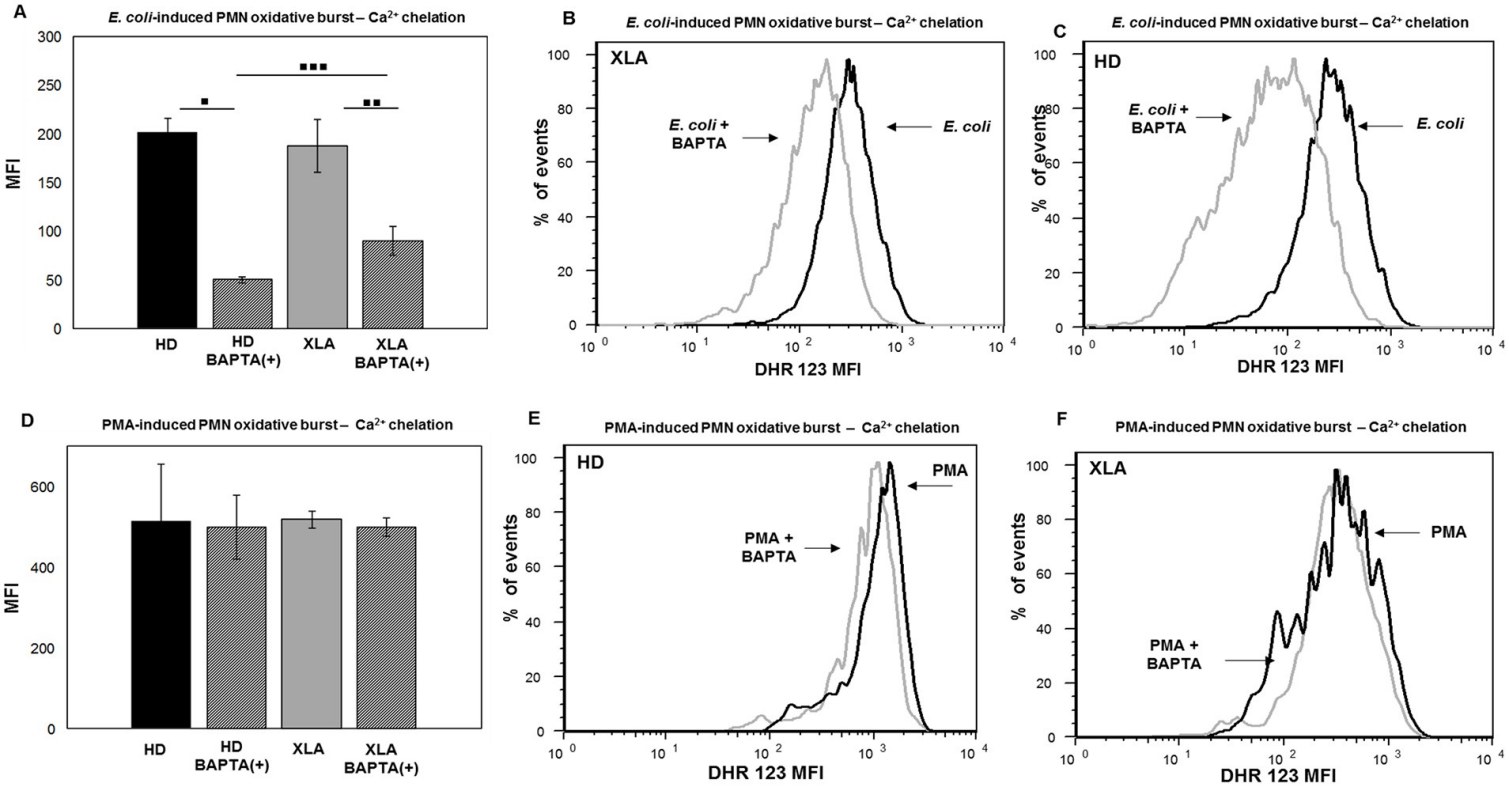
PMN oxidative burst—Ca^2+^ chelation. Whole blood samples were pre-treated with 100 μM of the calcium chelator BAPTA-AM for 30 min and incubated in a water bath for 20 min at 37°C with opsonized *E*. *coli* (1-2x10^9^/ml) and PMA (1.62 mM). The conversion of DHR 123 to R 123 was used to evaluate the intracellular ROS production. FSC and SSC characteristics were used to identify the PMN population. (A) BAPTA-AM treatment cause a stronger reduction of *E*. *coli*-induced oxidative burst in HD than XLA (▪▪▪p = 0.003). (A) The average reduction in HD was about 75% (▪p = 0.01) and it was about 50% in XLA patients (▪▪p = 0.02). (D) BAPTA-AM did not affect the oxidative burst induced by PMA both HD and XLA patients. Results are expressed as Mean Fluorescence Intensity. Histograms denote mean values and bars standard deviation. Statistical significance, indicated as *p* value, was determined by the nonparametric Mann-Whitney test and Wilcoxon Signed Rank test. **(**B and C) It shown PMN *E*. *coli-*induced oxidative burst in a representative HD and XLA patient, black peak BAPTA-AM(-) and gray peak BAPTA-AM(+). (E and F) It shown PMN PMA-induced oxidative burst in a representative HD and XLA patient, black peak BAPTA-AM(-) and gray peak BAPTA-AM(+).

*E*. *coli*-induced oxidative burst by monocytes was more inhibited by calcium chelation in HD than XLA (Fig 10A). The average reduction in XLA was about 50% (Fig 10B) while in HD was about 75% (Fig 10C). This finding suggests that in XLA patients the Ca^2+^ mobilization required for the ROS production was necessary but less critical than in HD. Also in PMN, BAPTA-AM induced a mild reduction of the *E*. *coli*-induced oxidative burst in XLA than HD (Fig 11A). The average reduction in XLA was about 50% (Fig 11B), while in HD was about 75% (Fig 11C). By contrast, the Ca^2+^ chelation did not affect PMN oxidative burst induced by PMA. Indeed, PMA induced a normal oxidative burst even when calcium was chelated (Fig 11D and 11F). This observation was expected since the PMA induces the ROS production through a direct binding to protein kinase C (PKC) [46, 47].

## Discussion

Mutations in BTK gene lead to an impaired B cell development resulting in absent or very low number of B-cells and profound hypogammaglobulinemia in patients affected by XLA [1, 2]. Previous studies on patients with XLA [7–9] showed the involvement of BTK in innate immune cell responses [48] and its role in human DC responses upon TLR9 engagement [49]. Moreover, studies on the immune function of XLA patients who lack functional BTK might also provide information concerning the consequences of drugs acting as BTK inhibitors on innate immunity of patients under these new treatments [50–54].

**Fig 12:**
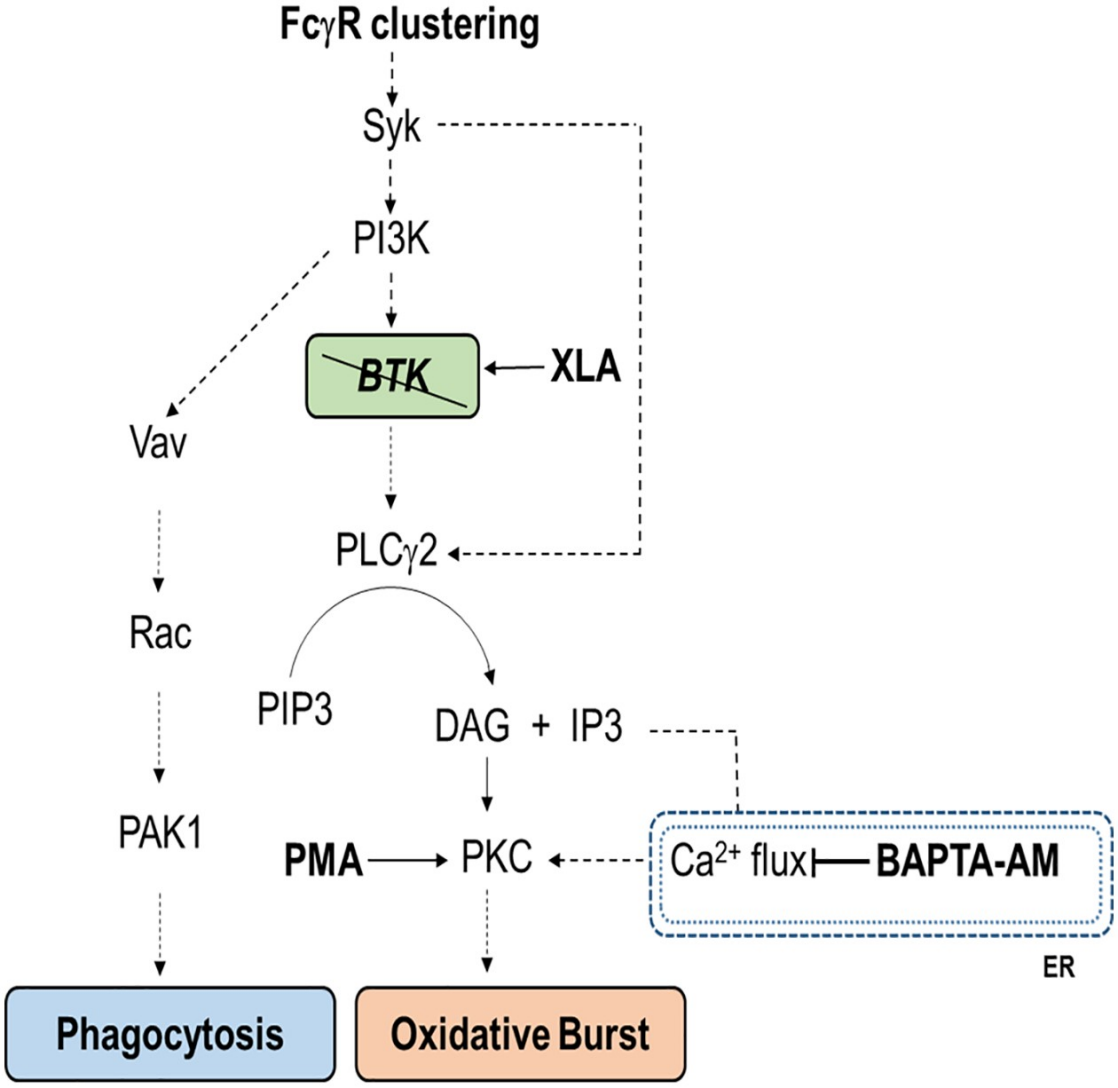
Schematic representation of signaling pathways for phagocytosis and oxidative burst. As a result of FcγR clustering, two separate pathways lead to phagocytosis, through a calcium independent way, or to the oxidative burst in a calcium dependent manner. Syk induce the activation of Vav and the downstream signaling that lead to the phagocytosis process. The main pathway for the activation of the oxidative burst consist in the activation of Btk by Syk and the consequent phosphorylation of PLCγ2. PLCγ2 activated produce DAG and IP3 from PIP3. DAG directly activate PKC while IP3 bind IP3R on endoplasmic reticulum inducing Ca^2+^ mobilization that lead to PKC activation and finally to the oxidative burst. The lack of BTK in XLA patients is effectively bypassed by the direct stimulation of PLCγ2 by Syk. The inhibition of Ca^2+^ mobilization by BAPTA-AM strongly reduce the oxidative burst. PMA is able to bind PKC to the same binding site of DAG and bypass both the production of DAG and the calcium flux from endoplasmic reticulum.

In this study, we show that monocyte and PMN from XLA patients normally express those receptors involved in their activation, suggesting that they did not exhibit an altered activation status. Despite the lack of BTK, monocytes and PMN rapidly overexpressed surface receptors after stimulation with opsonized bacteria, suggesting that BTK could be dispensable or bypassed in pathways triggered by FcγR clustering. Moreover, the study of the oxidative burst and phagocytosis provided us information on the killing ability of innate immune cells in XLA patients. In agreement with previous studies [44, 55], the phagocytosis and respiratory burst induced through FcγR were preserved in monocytes and PMN confirming that the BTK kinase activity is bypassed during the engagement of FcγR. These results also suggested that BTK might not be essential for Ca^2+^ mobilization, an event normally required for ROS production. Indeed, activated BTK induce a rapid Ca^2+^ release trough the phosphorylation of PLCγ2 that generates diacylglycerol (DAG) and inositol 1,4,5-trisphosphate (IP3). IP3 binds IP3 receptors and activates Ca^2+^-release channels on the endoplasmic reticulum (ER), triggering the calcium flux [14, 56]. Both DAG and Ca^2+^ are able to activate the PKC, the enzyme responsible for NADPH oxidase activation [46, 47]. The lack of BTK in XLA patients might be bypassed by an efficient activation of PLCγ2 performed by Syk, as we show in Fig 12 [57]. In monocytes, PLCγ2 is activated by FcγRI and FcγRIIA whereas in neutrophils the engagement of FcγRIIA is sufficient for its activation [57]. Moreover, we found that BAPTA-AM exerted a greater inhibition of the oxidative burst in HD than XLA, suggesting that ROS production in XLA patients is less dependent on Ca^2+^ mobilization. It is possible that the residual ability to produce ROS in XLA patients depend on a greater efficacy of DAG-dependent stimulation of PKC, an enzyme normally maintained in an inactive conformation [58–60]. In agreement with this finding, we showed that the Ca^2+^ chelator BAPTA-AM did not affect the ROS production when PMA was provided as stimulus since phorbol esters are able to bind PKC in the same binding site of DAG and thus inducing kinase activation [46, 47, 61]. Our observations are supported by others studies reporting different pathways for Ca^2+^ mobilization [62–64] and are also in agreement with the conclusions made by Ren *et al* [36] who hypothesized that the inhibition of BTK kinase activity within a whole organism could have limited effects on the FcγR-mediated processes. Our results are also in agreement with the study of Marron *et al* [44] who showed a ROS production not augmented or compromised in neutrophils from patients with XLA but in contrast with the results of Honda *et al* [9] who described an excessive ROS production. One possibility that could explain these discrepancies is related to differences in the methods of PMN collection and in the stimuli used during the experimental procedures, in that we used a more physiological stimulus such as the opsonized *E*. *coli* or a higher PMA concentration. Moreover, we found that the FcγR-mediated phagocytosis performed by monocytes and PMN was fully functional and it remained unaltered when monocytes and PMN were pre-treated with a Ca^2+^ chelator, confirming the independence of phagocytosis by Ca^2+^ mobilization [65–68]. Thus, the BTK kinase activity or the Ca^2+^ mobilization downstream BTK did not appear to be required for this process, as previously shown using a BTK inhibitor [36].

The only abnormality found was the overexpression of CD66b, suggesting an hyperactivation status of PMN. CD66b is able to stimulate neutrophils and its crosslinking is able to induce the secretion of preformed interleukin-8 (IL-8) [69]. The dosage of the IL-8 in the plasma of patients was normal in unstimulated condition. After stimulation with *E*. *coli*, IL-8 showed a rapid increase, suggesting that PMN of XLA patients effectively respond to opsonized bacteria in terms of cytokine secretion. However, our data show that the altered expression of CD66b on PMN surface did not correlate with IL-8 and elastase secretion and therefore it needs to be better clarified.

Also others functions involved in functionality of PMN, such as migration and elastase degranulation, were well preserved in XLA patients, confirming that all the fundamental steps involved in PMN killing ability were fully functional.

XLA patients received a long-life substitutive treatment with polyvalent IgG as a prophylaxis against infections [20, 21]. IVIg infusions could exert an anti-inflammatory function, even if administrated at replacement dosages, acting on monocytes as we recently showed in monocyte from CVID [23]. Interestingly, in line with our previous observation, XLA patients showed an increased percentage of intermediate monocyte, possibly caused by their inflammatory condition. This observation suggest that a greater frequency of intermediate monocytes could be a common feature of different PAD. The prompt reduction of the number of intermediate monocytes observed after IVIg is in line with the observation made in CVID patients [23] confirming the normalizing effect of IVIg infusion on monocytes. In addition, we show that the *in vivo* IVIg administration did not affect the expression of any surface receptors considered. Consistent with our previous study [23], we show that IVIg administration induced a decrease of monocytes’ oxidative burst and phagocytosis. We hypothesize that the transient binding of IVIg to FcγR might reduce the availability of monocyte receptors for the binding with opsonized *E*. *coli*, resulting in a slight reduction of phagocytosis and consequently in a reduced ROS production.

In summary, the lack of BTK kinase activity in XLA did not affect monocytes and PMN functions. IVIg infusion exerted a dampening effect on monocytes compartment by reducing the pro-inflammatory subset and by reducing their killing ability.

## Supporting information

S1 Fig Identification of PMN population. Whole blood samples were treated to lyse red blood cells for 20 minutes at room temperature and washed twice. White cells were suspended in ice-cold PBS and stained at 4°C for 30 min with CD15 fluorochrome-labeled antibody. Samples were washed, suspended in ice-cold PBS and analyzed by flow cytometry. PMN were identified by Side Scatter (SSC) and CD15 fluorochrome-labeled antibody. CD15 was used as a specific marker of neutrophils. A representative XLA patient is shown A and B.
(PDF)

S2 Fig Correlation between oxidative burst and phagocytosis before and after IVIg. Figure shows a positive correlation between oxidative burst and phagocytosis confirming the dependence of oxidative burst process from phagocytosis. Correlation was calculated by means of linear regression analysis.
(PDF)

S1 Table Demographic and clinical data of patients XLA. *Serum IgG, IgA and IgM values refer to pre-infusion levels. M, male. COPD: Chronic obstructive pulmonary disease.
(PDF)

## Abbreviations

BAPTA-AM: 1,2-bis (2-aminophenoxy) ethane-N,N,N’,N’ tetraacetic acid tetrakis(acetoxymethyl ester)
BTK: Bruton's tyrosine kinase
DAG: diacylglycerol
fLMP: N-formyl-Met-Leu-Phe
HD: Healthy Donors
IVIg: Intravenous immunoglobulins
MFI: Mean Fluorescence Intensity
PAD: primary antibody deficiencies
PMA: phorbol 12-myristate-13-acetate
PMN: polymorphonuclear neutrophils
ROS: reactive oxygen species
Siglec 9: sialic acid-binding immunoglobulin-like lectin receptor
XLA: X-linked agammaglobulinemia
