# Supplementary figures and images for: The lack of BTK does not impair monocytes and polymorphonuclear cells functions in X-linked agammaglobulinemia under treatment with intravenous immunoglobulin replacement
Source: PLoS One. 2017 Apr 19;12(4):e0175961. doi: 10.1371/journal.pone.0175961 (PMC5397035; doi:10.1371/journal.pone.0175961)

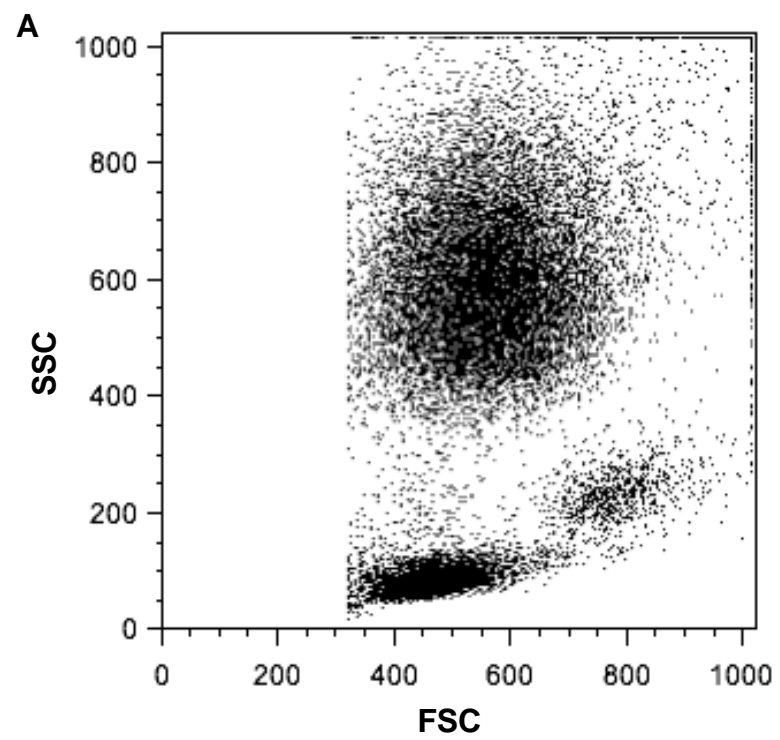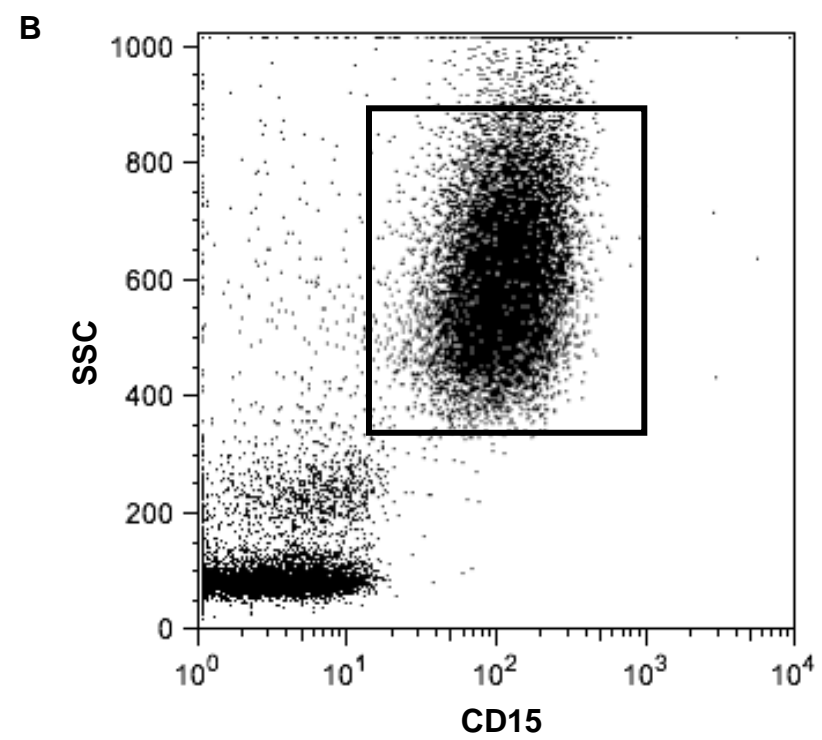

Supplement: S1 Fig — Whole blood samples were treated to lyse red blood cells for 20 minutes at room temperature and washed twice. White cells were suspended in ice-cold PBS and stained at 4°C for 30 min with CD15 fluorochrome-labeled antibody. Samples were washed, suspended in ice-cold PBS and analyzed by flow cytometry. PMN were identified by Side Scatter (SSC) and CD15 fluorochrome-labeled antibody. CD15 was used as a specific marker of neutrophils. A representative XLA patient is shown A and B. (PDF) [file pone.0175961.s001.pdf]

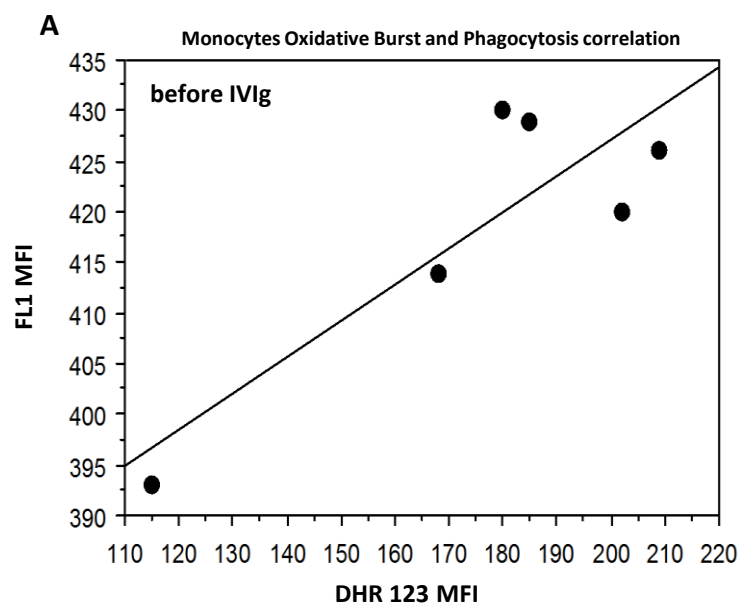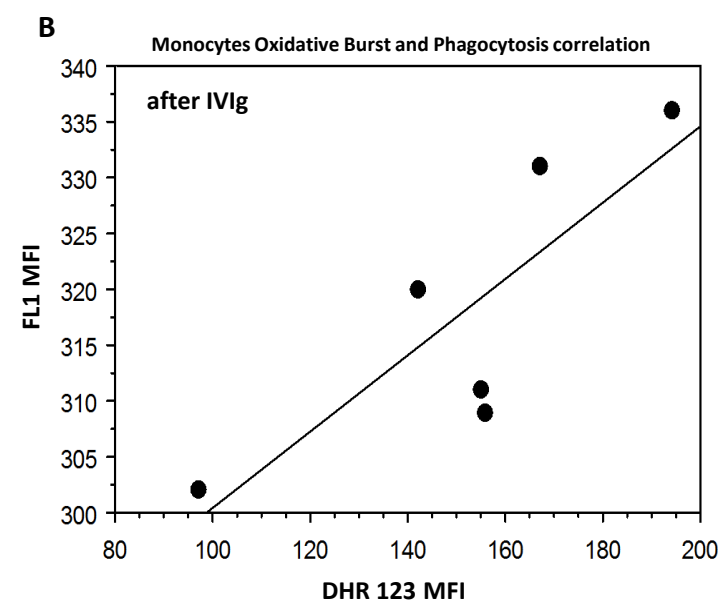

Supplement: S2 Fig — Figure shows a positive correlation between oxidative burst and phagocytosis confirming the dependence of oxidative burst process from phagocytosis. Correlation was calculated by means of linear regression analysis. (PDF) [file pone.0175961.s002.pdf]
