# Supplementary material for: The lack of BTK does not impair monocytes and polymorphonuclear cells functions in X-linked agammaglobulinemia under treatment with intravenous immunoglobulin replacement
Source: PLoS One. 2017 Apr 19;12(4):e0175961. doi: 10.1371/journal.pone.0175961 (PMC5397035; doi:10.1371/journal.pone.0175961)
Supplement: S1 Table — *Serum IgG, IgA and IgM values refer to pre-infusion levels. M, male. COPD: Chronic obstructive pulmonary disease. (PDF) [file pone.0175961.s003.pdf]

Patient	Age/sex	*Serum IgG (mg/dL)	*Serum IgA (mg/dL)	*Serum IgM (mg/dL)	B cells (%)	Monocytes (10^9/L)	Neutrophils (10^9/L)	Comorbidities
**1**	37/M	910	0.02	0.02	0.13	0.47	4.6	Celiac disease, respiratory tract infections, COPD
**2**	38/M	660	0.5	0.25	0.04	0.69	6.4	Respiratory tract infections, COPD
**3**	60/M	750	0.01	0.04	0.15	0.53	3.7	Epilepsy, stroke
**4**	39/M	680	0.03	0.02	0.2	0.48	3.8	Lymphadenomegaly, neuropathy, conjunctivitis, dermatitis
**5**	20/M	1090	0.02	0.01	0.2	0.92	6.1	Respiratory tract infections, COPD, dermatitis
**6**	26/M	240	0.02	0.01	0.1	0.93	5.3	Celiac disease, respiratory tract infections
